# Supplementary material for: lme4qtl: linear mixed models with flexible covariance structure for genetic studies of related individuals
Source: BMC Bioinformatics. 2018 Feb 27;19:68. doi: 10.1186/s12859-018-2057-x (PMC5830078; doi:10.1186/s12859-018-2057-x)
Supplement: Supplementary file 1 — Supplementary Tables and Figures. Supplementary Note 1: R code to compare lme4qtl and pedigreemm R packages. Supplementary Note 2: Multi-trait and multi-environment linear mixed models. Supplementary Note 3: R code applied to the GAIT2 data. (PDF 1341 kb) [file 12859_2018_2057_MOESM1_ESM.pdf]

Supplementary material for "lme4qtl: linear mixed models with flexible covariance structure for genetic studies of related individuals"

Andrey Ziyatdinov [1,*], Miquel Vázquez-Santiago [2,3], Helena Brunel [2], Angel Martinez-Perez [2], Hugues Aschard [1,4,5] and Jose Manuel Soria [2,5]

November 22, 2017

[1]Department of Epidemiology, Harvard T.H. Chan School of Public Health, Boston, Massachusetts, United States of America
[2]Unitat de Genòmica de Malalties Complexes, Institut d'Investigació Biomèdica Sant Pau (IIB-Sant Pau), Barcelona, Spain
[3]Unitat d'Hemostàsia i Trombosi, Hospital de la Santa Creu i Sant Pau, Barcelona, Spain
[4]Centre de Bioinformatique, Biostatistique et Biologie Intégrative (C3BI), Institut Pasteur, Paris, France
[5]Shared senior authorship

Contents

- Supplementary Tables and Figures
- Supplementary Note 1: R code to compare lme4qtl and pedigreemm R packages
- Supplementary Note 2: Multi-trait and multi-environment linear mixed models
- Supplementary Note 3: R code applied to the GAIT2 data

*to whom correspondence should be addressed

Supplementary Tables and Figures

Supplementary Table 1

Feature	lme4qtl	SOLAR	ASReml	GEMMA
Covariance for random effects	✓	✓	✓	✓
Covariance for residuals	(1)	✓	✓	✗
Methods for sparse covariances	✓	✗	✓	✗
Methods for dense covariances	(2)	✓	✓	✓
More than one covariances	✓	✓	✓	✗
Restriction on parameters	✓	✗	✗	✗
Gene-by-environment design	✓	(3)	✓	✗
Longitudinal design	✓	(3)	✓	✗
Free software	✓	✓	✗	✓
Open source	✓	✗	✗	✓

Table 1: Comparison among lme4qtl and selected stand-alone tools for genetic association analysis: SOLAR [1], ASReml [2] and GEMMA [3]. Notes: (1) The lme4qtl packages does not support any structures of residual variance, as the lme4 package does not has this feature yet. However, we showed two *ad hoc* solutions in Supplementary Note 3. (2) The lme4qtl package is based on sparse matrix methods from the lme4 package. In principle, dense matrix operations are still possible, but that might lead to considerable overhead in computation resources, as presented in Supplementary Figure 4 and discussed in the main text. (3) SOLAR requires specific tcl scripts (not publicly available) to parametrize either gene-by-environment or longitudinal models.

Supplementary Table 2

Feature	lme4qtl	pedigreemm	lmekin	Gaston	regress	rrBLUP
Extension of lme4	✓	✓	✗	✗	✗	✗
Covariance for random effects	✓	(1)	✓	✓	✓	✓
Covariance for residuals	(2)	(2)	✓	✗	✗	✗
Methods for sparse covariances	✓	✓	✓	✗	✗	✗
Methods for dense covariances	(3)	(3)	✓	✓	✓	✓
More than one covariances	✓	✓	✓	✓	✓	✗
Restriction on parameters	✓	✗	✗	✗	✗	✗

Table 2: Comparison among lme4qtl and other selected R packages that implement linear mixed models and can be used in genetic studies: pedigreemm [4] that extends lme4 [5], lmekin function in the R package coxme [6], Gaston [7], regress [8] and rrBLUP [9]. Notes: (1) The pedigreemm package does not support custom covariances, but allows to define relationship matrices based on the pedigree information. (2) Both lme4qtl and pedigreemm packages do not support any structures of residual variance, as the lme4 package does not has this feature yet. However, we showed two *ad hoc* solutions in Supplementary Notes 2 and 3. (3) Both lme4qtl and pedigreemm packages are based on sparse matrix methods from the lme4 package. In principle, dense matrix operations are still possible, but that might lead to considerable overhead in computation resources, as presented in Supplementary Figure 4 and discussed in the main text.

Supplementary Table 3

Model	Fast SOLAR, hours	SOLAR, hours	*lme4qtl*, hours
`aptt ~ age + sex + (1|id)`	**3.8**	16.8	6.6
`aptt ~ age + sex + (1|hhid) + (1|id)`	—	25.1	**7.6**
`aptt ~ age + sex + (1|hhid) + (1|id) + (1|id7)`	—	27.9	**23.3**

Table 3: We performed several genome-wide screenings of the activated partial thromboplastin time (APTT) phenotype in the GAIT2 data [10]. We considered three types of models and compared the computation time between our software *lme4qtl* and SOLAR [1]. The three models differed in the number of random effects: a single genetic additive effect (expressed in the model formula as `(1|id)`); two house-hold and genetic additive effect (`(1|hhid) + (1|id)`); and three house-hold, genetic additive and dominance effects (`(1|hhid) + (1|id) + (1|id7)`). The GAIT2 study included 903 individuals (those with measured values of APTT) in 35 extended families. The number of tested genetic markers consisted of 263,764 SNPs and indels, which passed the minimum allele frequency threshold of 1%. The analysis was performed on a desktop computer (2.8GHz quad-core Intel Core i5 processor, 8GB RAM).

Supplementary Figures

Supplementary Figure 1

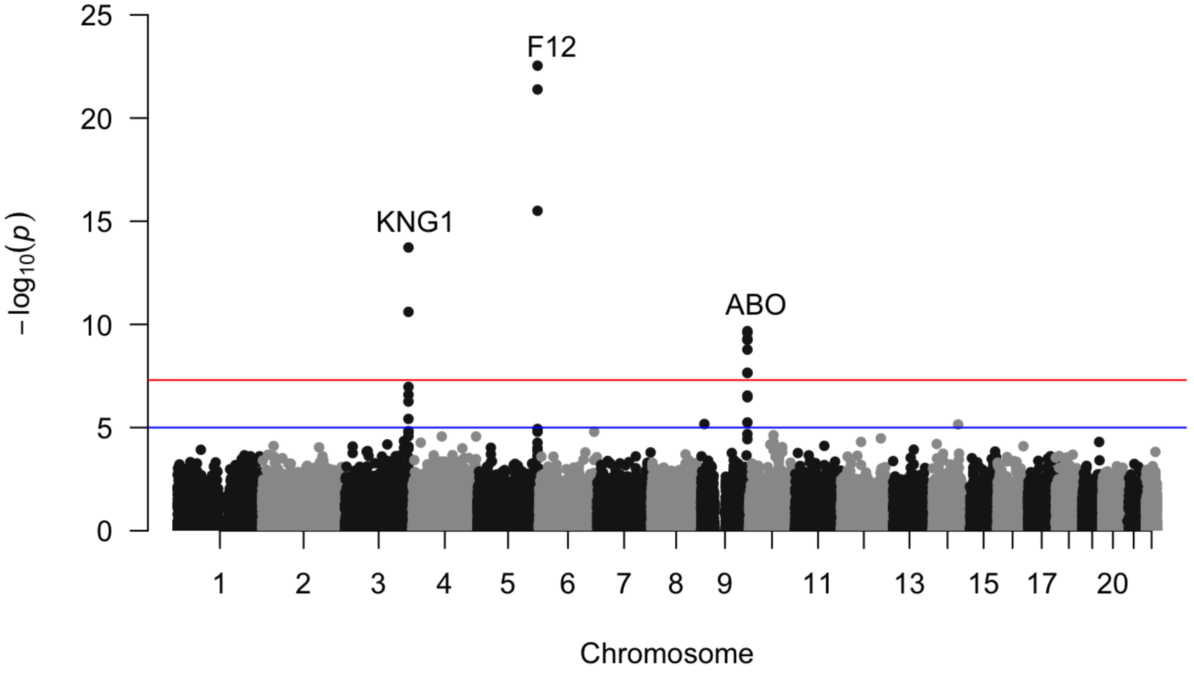

Figure 1: Genome-wide association study of APTT in the GAIT2 data computed by the *lme4qtl* R package partially replicates previously reported loci in a larger cohort of 9,240 individuals [11]. Three loci, in genes *KNG1*, *F12* and *ABO*, passed the genome-wide significant threshold at 5×10^{-8}, depicted as red horizontal line on the plot.

Supplementary Figure 2

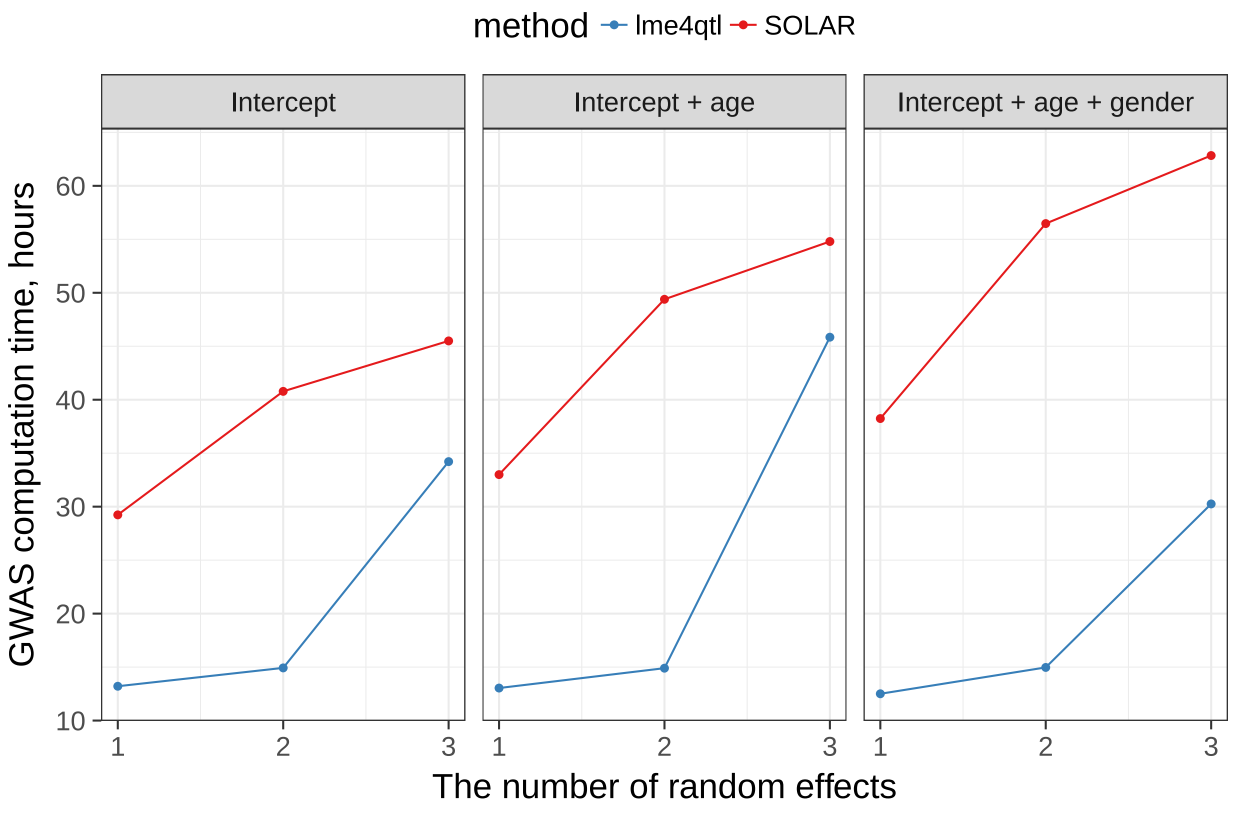

Figure 2: The plot represents the computation times reported in Table 3 on right panel. Other left and central panels show the results for the same experiment as in Table 3, but the list of fixed effects is less, either one (the intercept) or two (the intercept and age).

Supplementary Figure 3

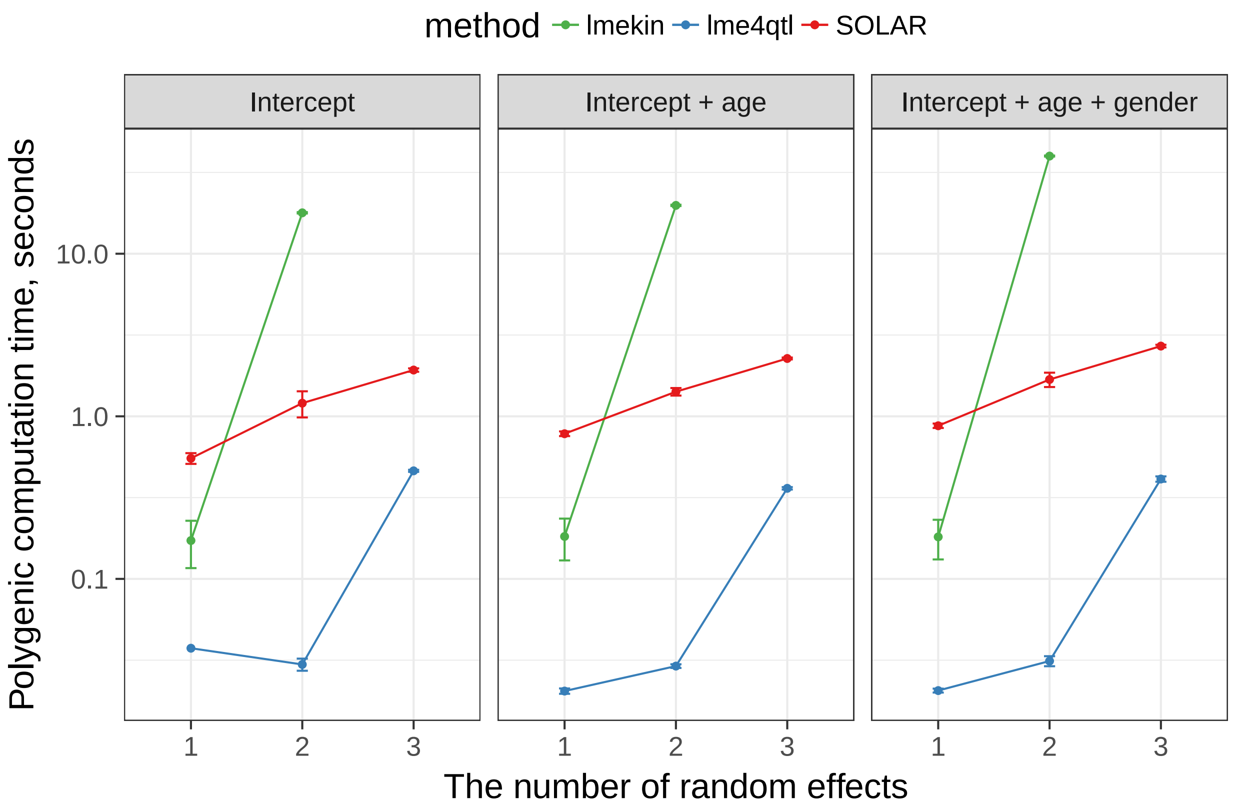

Figure 3: Comparison in computation time among three tools – our software *lme4qtl*, SOLAR [1] and *lmekin* [6] – showed the fastest performance of *lme4qtl*. We fitted a polygenic model of the activated partial thromboplastin time (APTT) phenotype measured in the GAIT2 data [10]. Models were different in the number of random effects: a single genetic additive effect (expressed in the model formula as `(1|id)`); two house-hold and genetic additive effect (`(1|hhid) + (1|id)`); and three house-hold, genetic additive and dominance effects (`(1|hhid) + (1|id) + (1|id7)`). Models also were different in the number of fixed effects (covariates): a single covariate (`Intercept`), two covariates (`Intercept + age`) and three covariates (`Intercept + age + gender`). The GAIT2 study included 903 individuals (those with measured values of APTT) in 35 extended families. The analysis was performed on a desktop computer (2.8GHz quad-core Intel Core i5 processor, 8GB RAM). We repeated each measurement of computational time 10 times and reported the mean value and its standard error in the figure. The numbers on y axis are base-10 log scaled.

Supplementary Figure 4

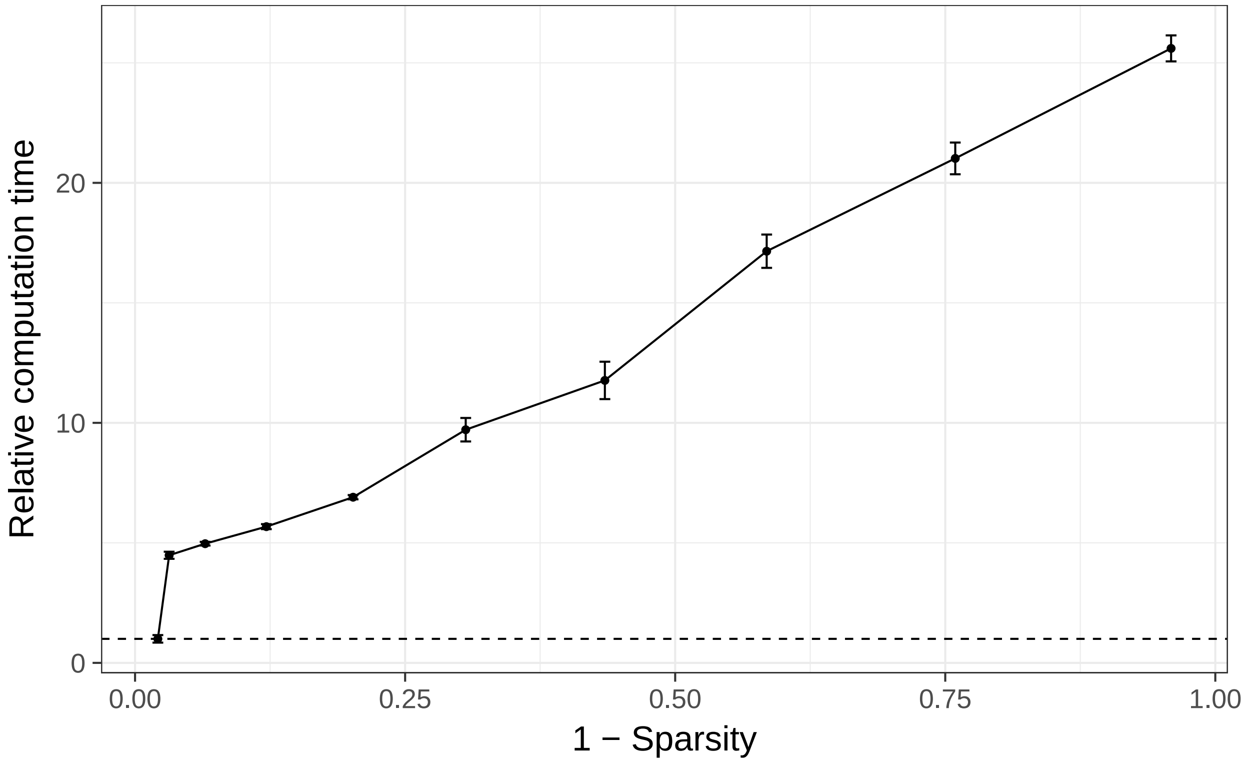

Figure 4: The computation time of polygenic model (devided by the number of interations in the optimization algorithm) fitted by *lme4qtl* increases as the sparsity of the genetic relatedness matrix is reduced, since the *lme4* machinery is optimized for linear algebra operations on sparse matrices. The sparsity is measured as the proportion of zero entries in the relatedness matrix (`mat`). Point on the plot with the lowest sparsity corresponds to a model fitted with the original GAIT2 genetic additive relatedness matrix. The dashed line marks the reference computation time. Other points come from models fitted with modified matrices varying their sparsity. The polygenic model is estimated for the activated partial thromboplastin time (APTT) measured in 903 individuals from the family-based GAIT2 study. The R code used for computation was `relmatLmer(aptt ~ age + gender + (1|id), dat, relmat = list(ID = mat))`. The analysis was performed on a desktop computer (2.8GHz quad-core Intel Core i5 processor, 8GB RAM). We repeated each measurement of computational time 10 times and reported the mean value and its standard error in the figure.

References

[1] L Almasy and J Blangero. Multipoint quantitative-trait linkage analysis in general pedigrees. *American journal of human genetics*, 62(5):1198–211, May 1998.

[2] Arthur R Gilmour, BJ Gogel, BR Cullis, R Thompson, D Butler, et al. *ASReml user guide release 3.0*, 2009. VSN International Ltd, Hemel Hempstead, UK.

[3] Xiang Zhou and Matthew Stephens. Genome-wide efficient mixed-model analysis for association studies. *Nature genetics*, 44(7):821–824, 2012.

[4] AI Vazquez, DM Bates, GJM Rosa, D Gianola, and KA Weigel. Technical note: an r package for fitting generalized linear mixed models in animal breeding. *Journal of animal science*, 88(2):497–504, 2010.

[5] Douglas Bates, Martin Mächler, Ben Bolker, and Steve Walker. Fitting linear mixed-effects models using lme4. *Journal of Statistical Software*, 67(1):1–48, 2015.

[6] Terry M. Therneau. *coxme: Mixed Effects Cox Models*, 2015. R package version 2.2-5.

[7] Hervé Perdry and Claire Dandine-Roulland. *Gaston: Genetic Data Handling (QC, GRM, LD, PCA) & Linear Mixed Models*, 2017. R package version 1.5.

[8] David Clifford and Peter McCullagh. *The regress package*, 2014. R package version 1.3-15.

[9] Jeffrey B Endelman. Ridge regression and other kernels for genomic selection with r package rrblup. *The Plant Genome*, 4(3):250–255, 2011.

[10] Laura Martin-Fernandez, Andrey Ziyatdinov, Marina Carrasco, Juan Antonio Millon, Angel Martinez-Perez, Noelia Vilalta, Helena Brunel, Montserrat Font, Anders Hamsten, Juan Carlos Souto, et al. Genetic determinants of thrombin generation and their relation to venous thrombosis: results from the GAIT-2 project". *PloS one*, 11(1):e0146922, 2016.

[11] Weihong Tang, Christine Schwienbacher, Lorna M Lopez, Yoav Ben-Shlomo, Tiphaine Oudot-Mellakh, Andrew D Johnson, Nilesh J Samani, Saonli Basu, Martin Gögele, Gail Davies, et al. Genetic associations for activated partial thromboplastin time and prothrombin time, their gene expression profiles, and risk of coronary artery disease. *The American Journal of Human Genetics*, 91(1):152–162, 2012.

Supplementary Note 1: R code to compare lme4qtl and pedigreemm R packages

Contents

About 1

Include 1

Data 2
Covariance matrices . . . 2

Models 3
A single kinship matrix . . . 3
A single custom covariance matrix . . . 3
Rank deficiency . . . 4
A single kinship matrix + random slope . . . 5
Restriction on model parameters . . . 5
Two covariance matrices . . . 5

References 7

About

The R package `pedigreemm` was first in extending the `lme4` R package for particular applications in the animal breeding field (Vazquez et al. 2010). Custom covariance (genetic additive) matrix are defined using the pedigree annotation information (`pedigree` argument of `pedigreemm` function). Although the `lme4qtl` package borrows the same idea of `pedigreemm`, `lme4qtl` provides a larger list of genetic models that are *not* possible with `pedigreemm`. In particular, these models include:

- models with a single or several custom covariances (not necessary linked to pedigree information);
- models with random slopes and other similar models like gene-by-environment interaction models;
  - the restriction on model parameters, e.g. the correlation coefficient is zero, is supported.

Here, we show models that are available with `lme4qtl` and not with `pedigreemm`.

Include

First, we load R packages necessary for data analysis.

```
library(Matrix)
library(magrittr)
```

```
library(pedigreemm)

library(lme4qtl)
```

Data

We use an example data set `milk` from the `pedigreemm` package. See `?milk` for description.

Here, we work on a subset of this dataset (`milk_subset`) to reduce the computation time.

```
data(milk)

milk <- within(milk, {
  id <- as.character(id)
  sdMilk <- milk / sd(milk)
})

ids <- with(milk, id[sire %in% 1:3]) # for more ids: c(1:3, 319-321)
milk_subset <- subset(milk, id %in% ids)

milk_subset <- within(milk_subset, {
   herd <- droplevels(herd)
   herd_id <- paste0("herd", seq(1, length(herd)))
})
```

Covariance matrices

A mixed model we are going to fit will have two random effects, groupings based on two ID variables:

- `id`, a numeric identifier of cow (the genetic additive effect);
- `herd`, a factor indicating the herd (the shared environmental effect).

Further we derive the covariance matrices (among samples) due to these two random effects.

```
A_herd <- with(milk_subset, model.matrix(~ herd - 1)) %>%
  tcrossprod %>% Matrix

rownames(A_herd) <- milk_subset$herd_id
colnames(A_herd) <- milk_subset$herd_id
```

```
A_gen <- getA(pedCowsR)

stopifnot(all(ids %in% rownames(A_gen)))
ind <- rownames(A_gen) %in% ids

A_gen <- A_gen[ind, ind]
```

```
image(A_herd, main = "A_herd")
image(A_gen, main = "A_gen")
```

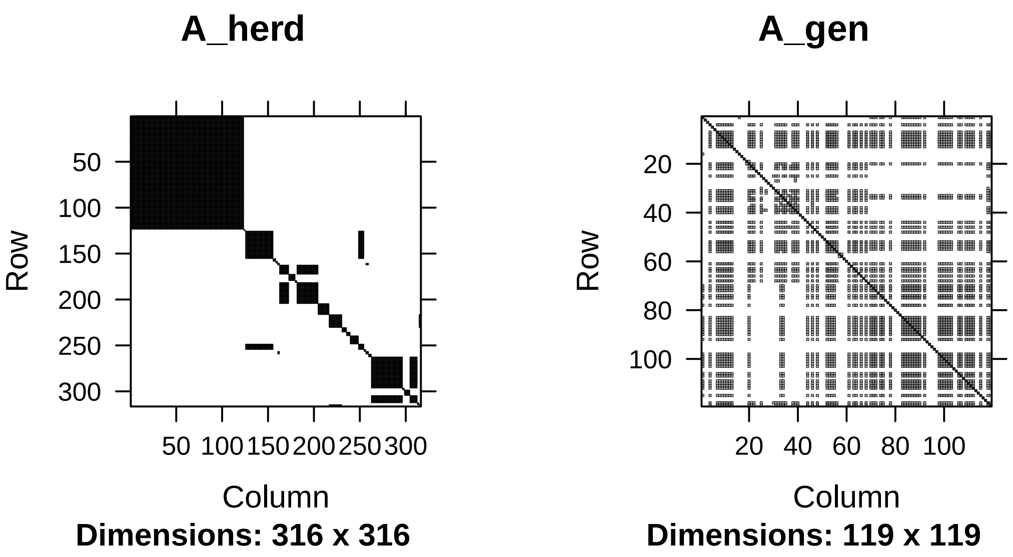

Models

A single kinship matrix

Both packages can fit a basic model with a single genetic effect, for which the `pedigreemm` R package was sought.

```
m1_pmm <- pedigreemm(sdMilk ~ lact + log(dim) + (1|id) + (1|herd),
  data = milk_subset, pedigree = list(id = pedCowsR))

VarCorr(m1_pmm)
```

```
 Groups   Name        Std.Dev.
 id       (Intercept) 0.55436
 herd     (Intercept) 0.55630
 Residual             0.59894
```

```
m1_relmat <- relmatLmer(sdMilk ~ lact + log(dim) + (1|id) + (1|herd),
  data = milk_subset, relmat = list(id = A_gen))

VarCorr(m1_relmat)
```

```
 Groups   Name        Std.Dev.
 id       (Intercept) 0.55436
 herd     (Intercept) 0.55630
 Residual             0.59894
```

We see that the estimation of variance components from both packages are identical.

A single custom covariance matrix

`lme4qtl` packages allows for custom covariance matrices, while `pedigreemm` does not.

```
m2_lmer <- lmer(sdMilk ~ (1|herd), milk_subset)
VarCorr(m2_lmer)
```

```
 Groups   Name        Std.Dev.
 herd     (Intercept) 0.54833
 Residual             0.81060
```

```r
m2_relmat <- relmatLmer(sdMilk ~ (1|herd_id), milk_subset,
  relmat = list(herd_id = A_herd))
VarCorr(m2_relmat)
```

```
 Groups   Name        Std.Dev.
 herd_id  (Intercept) 0.54833
 Residual             0.81060
```

```r
(try(m2_pmm <- pedigreemm(sdMilk ~ (1|herd_id), milk_subset,
  pedigree = list(herd_id = A_herd))))
```

```
[1] "Error : all(sapply(pedigree, is, class2 = \"pedigree\")) is not TRUE\n"
attr(,"class")
[1] "try-error"
attr(,"condition")
<simpleError: all(sapply(pedigree, is, class2 = "pedigree")) is not TRUE>
```

```r
getME(m2_lmer, "Ztlist")[[1]] %>% crossprod %>% image
getME(m2_relmat, "Ztlist")[[1]] %>% crossprod %>% image
```

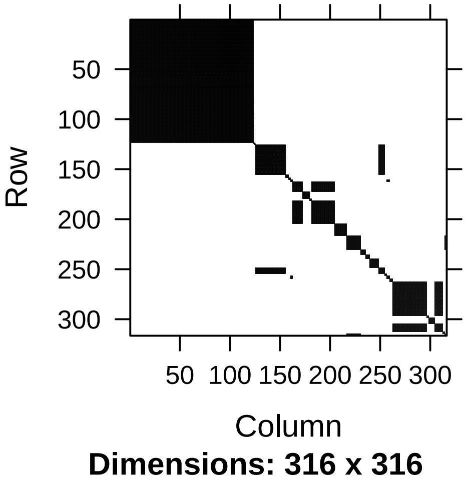

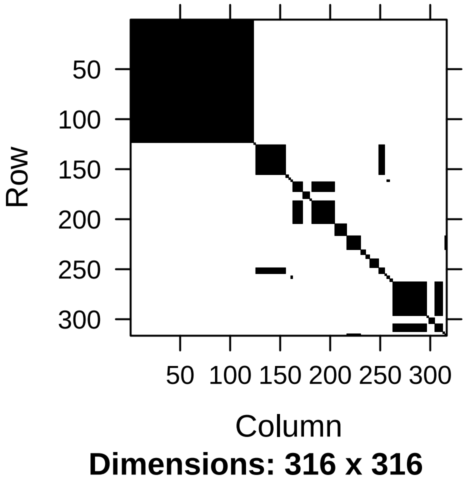

Rank deficiency

`A_herd` is a low-rank matrix, but `lme4qtl` is able to deal with this rank deficiency situation by replacing the Cholesky decomposition by the EVD operation. The `pedigreemm` package only uses the Cholesky decomposition.

```r
A_herd %>% dim
```

```
[1] 316 316
```

```r
A_herd %>% as.matrix %>% qr %$% rank
```

```
[1] 21
```

A single kinship matrix + random slope

Complex models are possible with `lme4qtl`, for example, those with a random slope effect.

```
m3_relmat <- relmatLmer(sdMilk ~ lact + log(dim) + (1 + lact|id) + (1|herd),
  data = subset(milk_subset, relmat = list(id = A_gen)))
VarCorr(m3_relmat)
```

```
 Groups   Name        Std.Dev. Corr
 id       (Intercept) 0.400268
          lact        0.094301 0.489
 herd     (Intercept) 0.593480
 Residual             0.585815
```

```
(try(m3_pmm <- pedigreemm(sdMilk ~ lact + log(dim) + (1 + lact|id) + (1|herd),
  data = milk_subset, pedigree = list(id = pedCowsR))))
```

```
[1] "Error in `levels<-`(`*tmp*`, value = if (nl == nL) as.character(labels) else paste0(labels,  : \n
attr(,"class")
[1] "try-error"
attr(,"condition")
<simpleError in `levels<-`(`*tmp*`, value = if (nl == nL) as.character(labels) else paste0(labels,
```

Restriction on model parameters

```
m3_relmat_rho0 <- relmatLmer(sdMilk ~ lact + log(dim) + (1 + lact|id) + (1|herd),
  data = subset(milk_subset, relmat = list(id = A_gen)),
  vcControl = list(rho0 = list(id = 2)))
VarCorr(m3_relmat_rho0)
```

```
 Groups   Name        Std.Dev. Corr
 id       (Intercept) 0.46014
          lact        0.11909  0.000
 herd     (Intercept) 0.58854
 Residual             0.57998
```

Two covariance matrices

```
m5 <- relmatLmer(sdMilk ~ (1|herd) + (1|id), milk_subset,
  relmat = list(id = A_gen))
VarCorr(m5)
```

```
 Groups   Name        Std.Dev.
 id       (Intercept) 0.54054
 herd     (Intercept) 0.54286
 Residual             0.64997
```

```
m6 <- relmatLmer(sdMilk ~ (1|herd_id) + (1|id), milk_subset,
  relmat = list(herd_id = A_herd, id = A_gen))
VarCorr(m6)
```

```
 Groups   Name        Std.Dev.
 herd_id  (Intercept) 0.54286
 id       (Intercept) 0.54054
```

```
 Residual              0.64997
```

```r
getME(m5, "Ztlist")[[1]] %>% crossprod %>% image
getME(m6, "Ztlist")[[2]] %>% crossprod %>% image
```

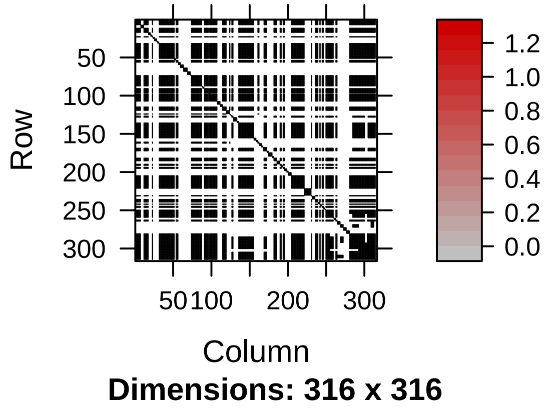

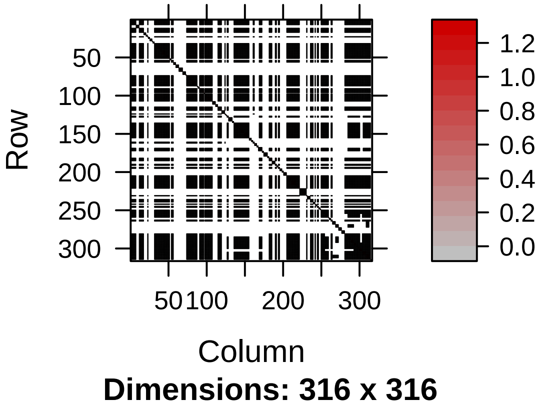


```r
getME(m5, "Ztlist")[[2]] %>% crossprod %>% image
getME(m6, "Ztlist")[[1]] %>% crossprod %>% image
```

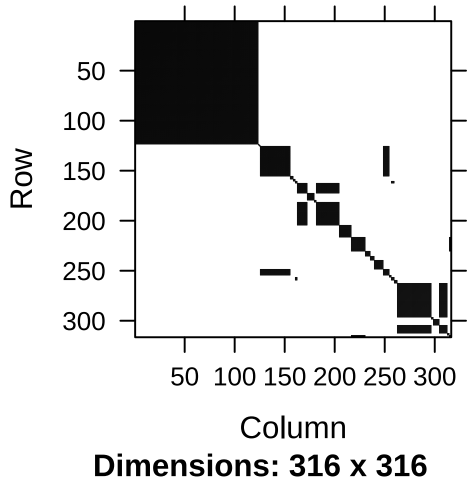

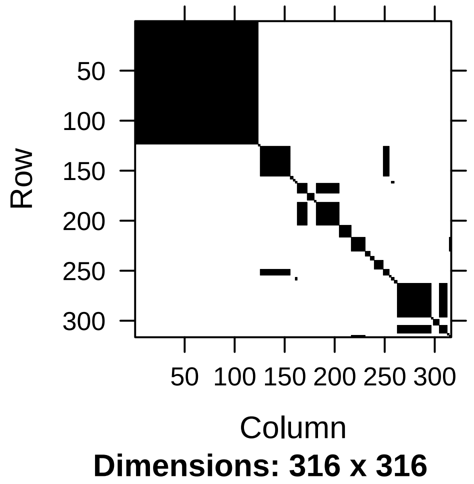


```r
(try(m3 <- pedigreemm(sdMilk ~ lact + log(dim) + (1|id) + (1|herd_id),
  data = milk_subset, pedigree = list(id = pedCowsR, herd_id = A_herd))))
```

```
[1] "Error : all(sapply(pedigree, is, class2 = \"pedigree\")) is not TRUE\n"
attr(,"class")
[1] "try-error"
attr(,"condition")
<simpleError: all(sapply(pedigree, is, class2 = "pedigree")) is not TRUE>
```

References

Vazquez, AI, DM Bates, GJM Rosa, D Gianola, and KA Weigel. 2010. "Technical Note: An R Package for Fitting Generalized Linear Mixed Models in Animal Breeding." *Journal of Animal Science* 88 (2). American Society of Animal Science: 497–504.

Supplementary Note 2: Multi-trait and multi-environment linear mixed models

We consider a simple polygenic model with two random effects, the additive genetic effect and the residual errors (also referred to as environment effect).

Single-trait linear mixed model

A linear model describes observations of a trait, measured in n individuals and stored in a vector $y_{n\times 1}$.

$$y = X\beta + Zu + e$$

where $X_{n\times p}$ and $Z_{n\times n}$ are incidence matrices, p is the number of fixed effects, $\beta_{p\times 1}$ is a vector of fixed effects, $u_{n\times 1}$ is a vector of a random polygenic effect, and $e_{n\times 1}$ is a vector of the residuals errors. The random vectors u and e are mutually uncorrelated and multivariate normally distributed, $\mathcal{N}(0, G_{n\times n})$ and $\mathcal{N}(0, R_{n\times n})$. The covariance matrices are parametrized with a few scalar parameters and have the form $G_{n\times n} = \sigma_g^2 A_{n\times n}$ and $R_{n\times n} = \sigma_e^2 I_{n\times n}$, where A is a genetic additive relationship matrix and I is the identity matrix.

Multi-trait linear mixed model

The model for a single trait can be extended to a more general case of two and more traits by stacking observations from traits together [1].

A linear model describes observations in two traits, measured in n individuals and stored in a vector $y_{2n\times 1}$.

$$y = X\beta + Zu + e$$

where $X_{2n\times p}$ and $Z_{2n\times 2n}$ are incidence matrices, p is the number of fixed effects, $\beta_{p\times 1}$ is a vector of fixed effects, $u_{2n\times 1}$ is a vector of a random polygenic effect, and $e_{2n\times 1}$ is a vector of the residuals errors. The random vectors u and e are mutually uncorrelated and multivariate normally distributed, $\mathcal{N}(0, G_{2n\times 2n})$ and $\mathcal{N}(0, R_{2n\times 2n})$.

The variance-covariance matrices $G_{2n\times 2n}$ and $R_{2n\times 2n}$ have a block structure and can be represented using as the Kronecker operator.

$$G_{2n\times 2n} = C_{2\times 2} \otimes A_{n\times n} = \begin{pmatrix} c_{11}A & c_{12}A \\ c_{21}A & c_{21}A \end{pmatrix} = \begin{pmatrix} \sigma_1^2 A & \rho\sigma_1\sigma_2 A \\ \rho\sigma_1\sigma_2 A & \sigma_2^2 A \end{pmatrix}_g$$

$$R_{2n\times 2n} = E_{2\times 2} \otimes I_{n\times n} = \begin{pmatrix} e_{11}I & e_{12}I \\ e_{21}I & e_{21}I \end{pmatrix} = \begin{pmatrix} \sigma_1^2 I & \rho\sigma_1\sigma_2 I \\ \rho\sigma_1\sigma_2 I & \sigma_2^2 I \end{pmatrix}_e$$

The diagonal entries $\sigma_{g_1}^2$ and $\sigma_{g_2}^2$ in the symmetric matrix C are marginal genetic variances for each trait, and the off-diagonal entries $\rho_g\sigma_{g_1}\sigma_{g_2}$ are covariances between the traits. The environment covariance $R_{2n\times 2n}$ is represented similarly.

Multi-environment linear mixed model

If a trait is measured in two environments, the previous model for two different traits can be applied [2]. Thus, the diagonal entries $\sigma_{g_1}^2$ and $\sigma_{g_2}^2$ in the symmetric matrix C are marginal genetic variances for each of two environment, and the off-diagonal entries $\rho_g\sigma_{g_1}\sigma_{g_2}$ are covariances between the environments. The environment covariance $R_{2n\times 2n}$ has a similar interpretation.

Blangero proposed statistical tests for the null hypothesis of no gene-environment interaction based on the likelihood ratio statistic, when comparing the full model and a reduced model. The first null model assumes that the genetic variances are equal in the null model [2, p. 535]. The second null model assumes that the genetic correlation coefficient is equal to 1.

Following the *lme4* authors' guidelines [3, Section A.1], we implemented three types of restrictions: the correlation is zero ($\rho_g = 0$), the variances are equal ($\sigma_{g_1} = \sigma_{g_2}$), and the correlation is one ($\rho_g = 1$). These types of restrictions can be extended to more general cases with multiple environments.

Multi-environment linear mixed model: a special case of sex-specificity

A sex-specificity model is a special case of gene-environment interactions where individuals are measured in single environments [2, p. 530].

A linear model describes observations in a trait, measured in n individuals and stored in a vector $y_{n\times 1}$.

$$y = X\beta + Zu + e$$

where $X_{n\times p}$ and $Z_{n\times n}$ are incidence matrices, p is the number of fixed effects, $\beta_{p\times 1}$ is a vector of fixed effects, $u_{n\times 1}$ is a vector of a random polygenic effect, and $e_{n\times 1}$ is a vector of the residuals errors. The random vectors u and e are mutually uncorrelated and multivariate normally distributed, $\mathcal{N}(0, G_{n\times n})$ and $\mathcal{N}(0, R_{n\times n})$.

The variance-covariance matrices $G_{n\times n}$ and $R_{n\times n}$ have a block structure stratified by gender.

$$G_{n\times n} = \begin{pmatrix} \sigma_1^2 A_{11} & \rho\sigma_1\sigma_2 A_{12} \\ \rho\sigma_1\sigma_2 A_{21} & \sigma_2^2 A_{22} \end{pmatrix}_g$$

$$R_{n\times n} = \begin{pmatrix} \sigma_1^2 I & \rho\sigma_1\sigma_2 I \\ \rho\sigma_1\sigma_2 I & \sigma_2^2 I \end{pmatrix}_e$$

Matrices A_{11}, A_{12}, A_{21} and A_{22} are four blocks of the matrix A stratified by gender. For example, A_{11} is the genetic relationship matrix that corresponds to males. As A is symmetric, $A_{12} = A_{21}$.

Parameters $\sigma_{g_1}^2$ and $\sigma_{g_2}^2$ are marginal genetic variances in males and females, and parameter ρ_g is the genetic correlation coefficient between the two genders. The environment covariance parameters have a similar interpretation.

The correlation coefficient ρ_e is restricted to zero, so the sex-specificity model is identifiable [2].

$$R_{n\times n} = \begin{pmatrix} \sigma_1^2 I & 0 \\ 0 & \sigma_2^2 I \end{pmatrix}_e$$

Sex-specificity linear mixed model in the GAIT2 data

In the main text of the manuscript, we showed two basic models for the analysis of APTT in the GAIT2 data, polygenic and association. Here, we present an advanced model that assesses the sex-specificity in the APTT phenotype.

Before conducting the analysis, we stored phenotype, age, gender, individual `id`, house-hold `hhid` variables and SNPs in a table `dat`. The additive genetic relatedness matrix was estimated by SOLAR using the pedigree information and stored in a matrix `mat`. A polygenic model `m1` was fitted to the data as follows.

```
m1 <- relmatLmer(aptt ~ age + gender + (1|id), dat,
  relmat = list(id = mat))
```

To assess the hypothesis of sex-specificity [2] for APTT, our package allows to fit such a polygenic model `m3` with multiple levels of relatedness.

```
m3 <- relmatLmer(aptt ~ age + gender + (0 + gender|id) + (0 + gender|rid), dat,
  relmat = list(id = mat), vcControl = list(rho0 = list(rid = 5)),
  weights = rep(1e10, nrow(dat)))
```

The first genetic random effect, denoted as `(0 + gender|id)`, has three parameters σ_{g_1}, σ_{g_2} and ρ_g, as described in the previous section. The second residual random effect, denoted as `(0 + gender|rid)`, also has three parameters, but the correlation coefficient is restricted to zero as specified in the `vcControl` argument. This restriction is necessary because the model is a special case of gene-environment interactions where individuals are measured in single environments [2, p. 530]. The variable `rid` is a copy of `id`, and using large values in the last argument `weights` is an *ad hoc* solution to cancel the independent and identically distributed residual error. We note that the `m3` model can be fitted without the *ad hoc*.

```
m3 <- relmatLmer(aptt ~ age + gender + (0 + gender|id) + (0 + dummy(gender)|rid), dat,
  relmat = list(id = mat))
```

Once the evidence of the gene-environment interaction in `m3` is confirmed [2], a new association model `m4` can be considered for the GWAS, in which a SNP, for example, `rs1`, has both marginal and interaction terms with the `gender` variable.

```
m4 <- update(m3, . ~ . + rs1 + rs1:gender)
anova(m3, m4)
```

Implementation of restriction on model parameters

The R code used in the previous section to fit the sex-specificity model has a special use of the `vcControl` parameter, that defines the restriction on variance components.

```
m3 <- relmatLmer(aptt ~ age + gender + (0 + gender|id) + (0 + gender|rid), dat,
  relmat = list(id = mat), vcControl = list(rho0 = list(rid = 5)),
  weights = rep(1e10, nrow(dat)))
```

To understand how the `vcControl` argument works, we need to write the covariance structure of random effects (`(0 + gender|id)` and `(0 + gender|rid)` using its associated Cholesky decomposition [3, Appendix A.1, p. 44, formula (69)].

$$\begin{pmatrix} \sigma_1^2 & \rho\sigma_1\sigma_2 \\ \rho\sigma_1\sigma_2 & \sigma_2^2 \end{pmatrix}_g = \begin{pmatrix} \theta_1 & 0 \\ \theta_2 & \theta_3 \end{pmatrix} \begin{pmatrix} \theta_1 & \theta_2 \\ 0 & \theta_3 \end{pmatrix} = \begin{pmatrix} \theta_1^2 & \theta_1\theta_2 \\ \theta_1\theta_2 & \theta_2^2 + \theta_3^2 \end{pmatrix}$$

$$\begin{pmatrix} \sigma_1^2 & \rho\sigma_1\sigma_2 \\ \rho\sigma_1\sigma_2 & \sigma_2^2 \end{pmatrix}_e = \begin{pmatrix} \theta_4 & 0 \\ \theta_5 & \theta_6 \end{pmatrix} \begin{pmatrix} \theta_4 & \theta_5 \\ 0 & \theta_6 \end{pmatrix} = \begin{pmatrix} \theta_4^2 & \theta_4\theta_5 \\ \theta_4\theta_5 & \theta_5^2 + \theta_6^2 \end{pmatrix}$$

Now it is clear that the environmental correlation can be restricted to zero by setting $\theta_5 = 0$. Consequently, the value of the `vcControl` argument is `list(rho0 = list(rid = 5))`.

The following table shows more options of using `vcControl`.

Condition	Parameter restrictions	`vcControl` value
$\rho_g = 0$	$\theta_2 = 0$	`list(rho0 = list(id = 2))`
$\rho_g = 1$	$\theta_3 = 0$	`list(rho1 = list(id = 3))`
$(\sigma_1)_g = (\sigma_2)_g$	$\theta_1^2 = \theta_2^2 + \theta_3^2$	`list(vareq = list(id = c(1, 2, 3)))`
$\rho_g = 0$, $\rho_e = 0$	$\theta_2 = 0$, $\theta_5 = 0$	`list(rho0 = list(id = 2, rid = 5))`

The use of names such as `rho0`, `rho1` and `vareq` is required, as these names are bound to particular implementation (the second column of the table given above) in the body of the `relmatLmer` function.

References

[1] Michael Lynch, Bruce Walsh, et al. *Genetics and analysis of quantitative traits*, volume 1. Sinauer Sunderland, MA, 1998.

[2] John Blangero. Statistical genetic approaches to human adaptability. *Human biology*, 81(5):523–546, 2009.

[3] Douglas Bates, Martin Mächler, Ben Bolker, and Steve Walker. Fitting linear mixed-effects models using lme4. *Journal of Statistical Software*, 67(1):1–48, 2015.

Supplementary Note 3: R code applied to the GAIT2 data

Contents

Introduction **1**
Load packages . . . 1

Parameters **2**

Load data **2**
Phenotype data . . . 2
Covariance matrices . . . 3

Polygenic analysis **3**
Diagnostics . . . 4
Inference . . . 5
Inference for heritability . . . 6
Confidence interval . . . 6
Likelihood ratio tests (LRTs) . . . 7
Summary . . . 8

Polygenic sex-specificity analysis **8**
Summary . . . 9

Additional analyses **9**
Dominance effect in addition to additive genetic and house-hold effects . . . 9
Two fitting methods for gene-by-gender . . . 10

R session info **11**

Introduction

Load packages

We need a list of R package, including our *lme4qtl* package, to perform the analysis.

```
library(plyr)
library(dplyr)

library(Matrix)
library(gridExtra)

library(lme4)
library(boot)

library(lme4qtl)
```

The next two packages complement the *lme4* functionality with additional inference procedures.

```r
library(lmerTest)
```

```
##
## Attaching package: 'lmerTest'

## The following object is masked from 'package:lme4':
##
##     lmer

## The following object is masked from 'package:stats':
##
##     step
```

```r
library(RLRsim)
```

Parameters

The GAIT2 family-based sample consists of 934 individuals. Here, we use a small subset of 10 markers from Chromosome 22 in the association analysis.

```r
N <- 934

chr <- 22
M <- 10
```

Load data

We need the following R packages (not publicly available) to load the GAIT2 data.

```r
library(gait)
library(solarius)
```

Data variables include

- table of phenotypes `phen` with such variables as
  - `aptt` outcome, the activated partial thromboplastin time (APTT)
  - `gender` and `age` as covariates or fixed effect
  - `id`, the individual identifier
  - `famid`, the family identifier
  - `hhid`, the house-hold identifier (not the same as `famid`)
- `dkin`, the double kinship matrix (additive genetic effect)
- `delta7`, matrix of dominance genetic effect

Phenotype data

```r
dir_phen <- "~/Data/GAIT2/phen/"
dir_snp <- "~/Data/GAIT2/ncdf/"

phen <- gait2.phen(dir_phen, transforms = "tr1", id.alert = TRUE, traits = "tr1_APTT")
```

```r
phen <- rename(phen,
  aptt = tr1_APTT,
```

```r
  gender = SEXf, age = AGEc,
  id = ID, famid = FAMID, hhid = HHID)

phen <- mutate(phen, rid = id, id7 = id)
```

Covariance matrices

```r
dkin <- Matrix(solarKinship2(phen))
delta7 <- Matrix(solarKinship2(phen, coef = "d"))
```

The next plot depicts sub-matrices (first 50 individuals) of the genetic additive (left) and dominance (right) covariance matrices.

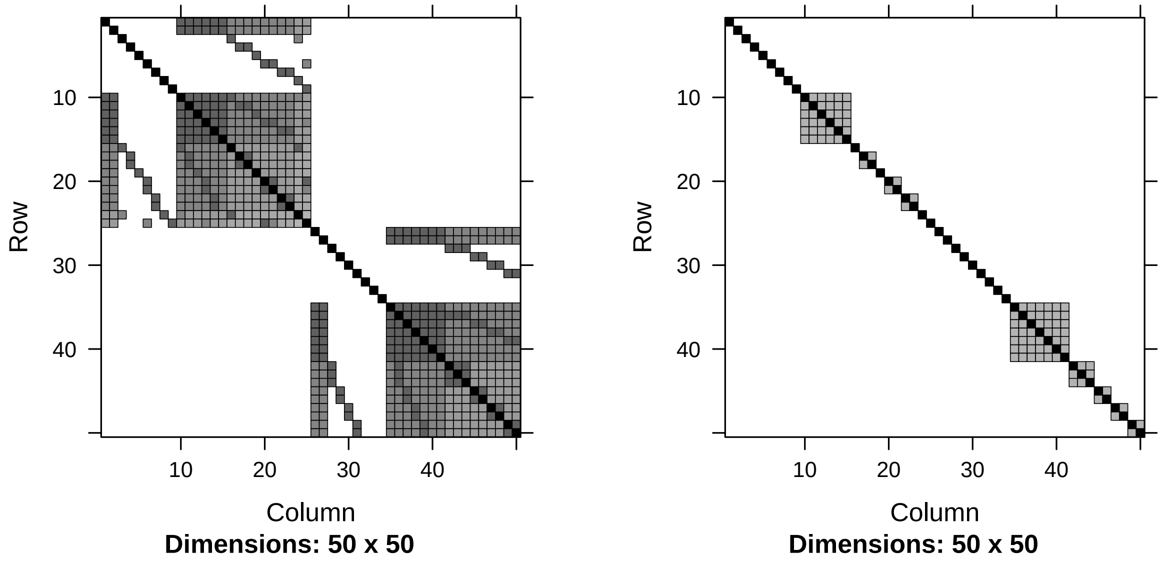

Polygenic analysis

The polygenic model of APTT has two random effects (apart from the residual variance), genetic additive and house-hold. In the case of the genetic effect, the covariance matrix `dkin` is introduced using `relmat` argument.

```r
m1 <- relmatLmer(aptt ~ age + gender + (1|id) + (1|hhid), phen, relmat = list(id = dkin))
m1
```

```
## Linear mixed model fit by REML ['lmerMod']
## Formula: aptt ~ age + gender + (1 | id) + (1 | hhid)
##    Data: phen
## REML criterion at convergence: 2355.299
## Random effects:
##  Groups   Name        Std.Dev.
##  id       (Intercept) 0.7270
##  hhid     (Intercept) 0.2582
##  Residual             0.5926
```

```
## Number of obs: 884, groups:  id, 884; hhid, 448
## Fixed Effects:
## (Intercept)          age      gender2
##     0.17759     -0.01687     -0.07376
```

Diagnostics

The residuals are expected to be normally distributed.

```r
r1 <- residuals(m1)
```

```r
qqnorm(r1)
qqline(r1)
```

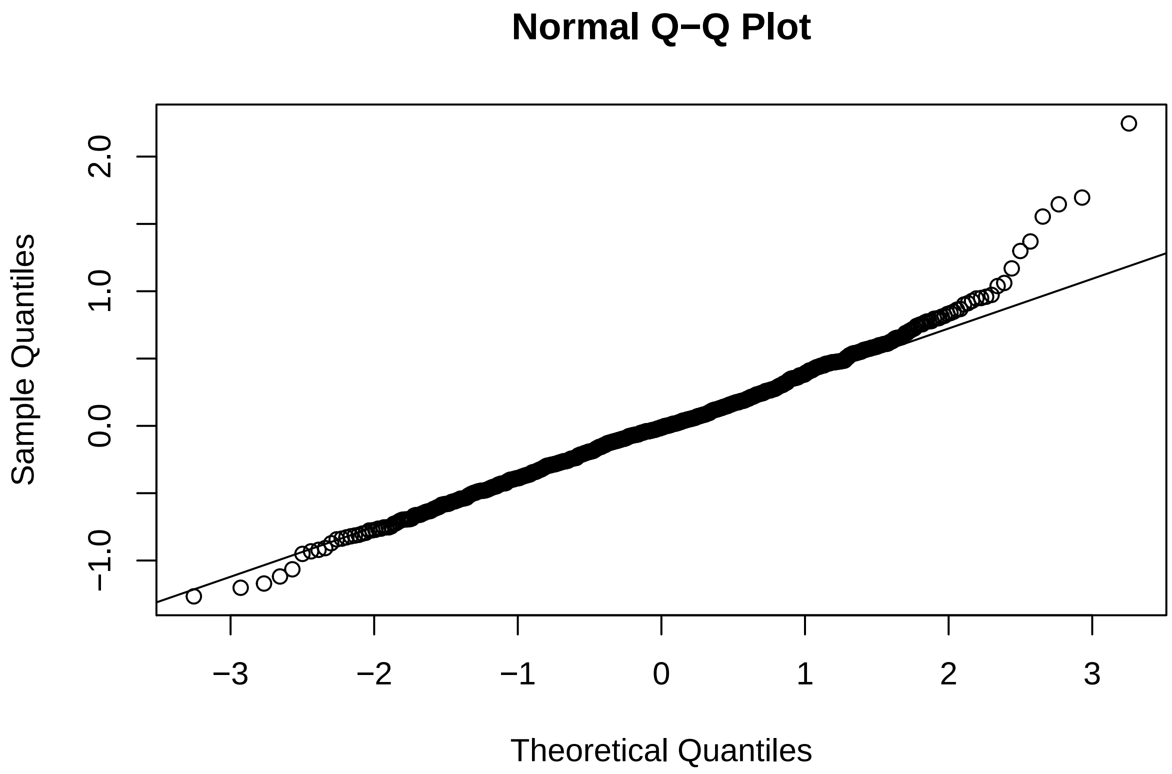


```r
hist(r1, breaks = 30)
```

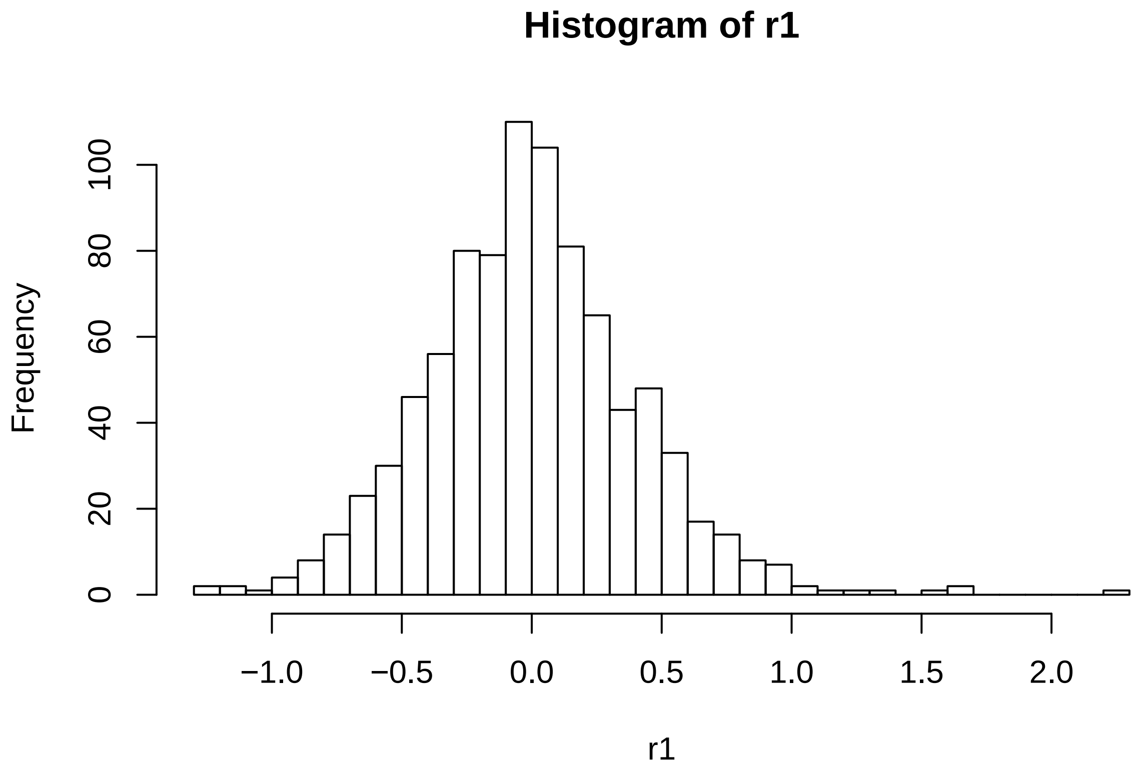

Inference

We use the `step` function from the R package `lmerTest`.

```
# `?lmerTest::step`
# the p-value thr. are set to 1 to disable terms dropping
step(m1, alpha.random = 1, alpha.fixed = 1)
```

```
##
## Random effects:
##      Chi.sq Chi.DF elim.num p.value
## id    70.44      1     kept  <1e-07
## hhid   2.98      1     kept  0.0842
##
## Fixed effects:
##         Sum Sq Mean Sq NumDF  DenDF  F.value elim.num Pr(>F)
## age    53.2671 53.2671     1 650.17 151.6804     kept <1e-07
## gender  0.5382  0.5382     1 794.99   1.5326     kept 0.2161
##
## Least squares means:
##           gender Estimate Standard Error  DF  t-value Lower CI Upper CI
## gender  1      1   0.1826         0.0586 459   3.1200    0.183    0.183
## gender  2      2   0.1088         0.0589 439   1.8500    0.109    0.109
##           p-value
## gender  1  0.0019 **
## gender  2  0.0655 .
```

```
## ---
## Signif. codes:  0 '***' 0.001 '**' 0.01 '*' 0.05 '.' 0.1 ' ' 1
##
##  Differences of LSMEANS:
##                Estimate Standard Error    DF t-value Lower CI Upper CI
## gender 1 - 2      0.1         0.0596 795.0    1.24   0.0738   0.0738
##                p-value
## gender 1 - 2     0.2
##
## Final model:
## relmat_lmer(formula = aptt ~ age + gender + (1 | id) + (1 | hhid),
##     data = phen, contrasts = list(gender = "contr.SAS"), relmat = list(id = dkin))
```

Inference for heritability

By definition, heritability is the proportion of explained variance.

```
vf <- as.data.frame(VarCorr(m1))[, c("grp", "vcov")]
vf$prop <- with(vf, vcov / sum(vcov))
```

grp	vcov	prop
id	0.53	0.56
hhid	0.07	0.07
Residual	0.35	0.37

Confidence interval

```
# `?lme4::profile`
prof <- profile(m1, which = "theta_", prof.scale = "varcov")
```

```
# `?lme4qtl::varpropProf`
prof_prop <- varpropProf(prof)
```

```
ci <- confint(prof_prop, level = 0.95)
ci
```

```
##                2.5 %     97.5 %
## .sigprop01 0.4450731 0.84293219
## .sigprop02 0.0000000 0.06472766
## .sigmaprop 0.1461354 0.50813557
```

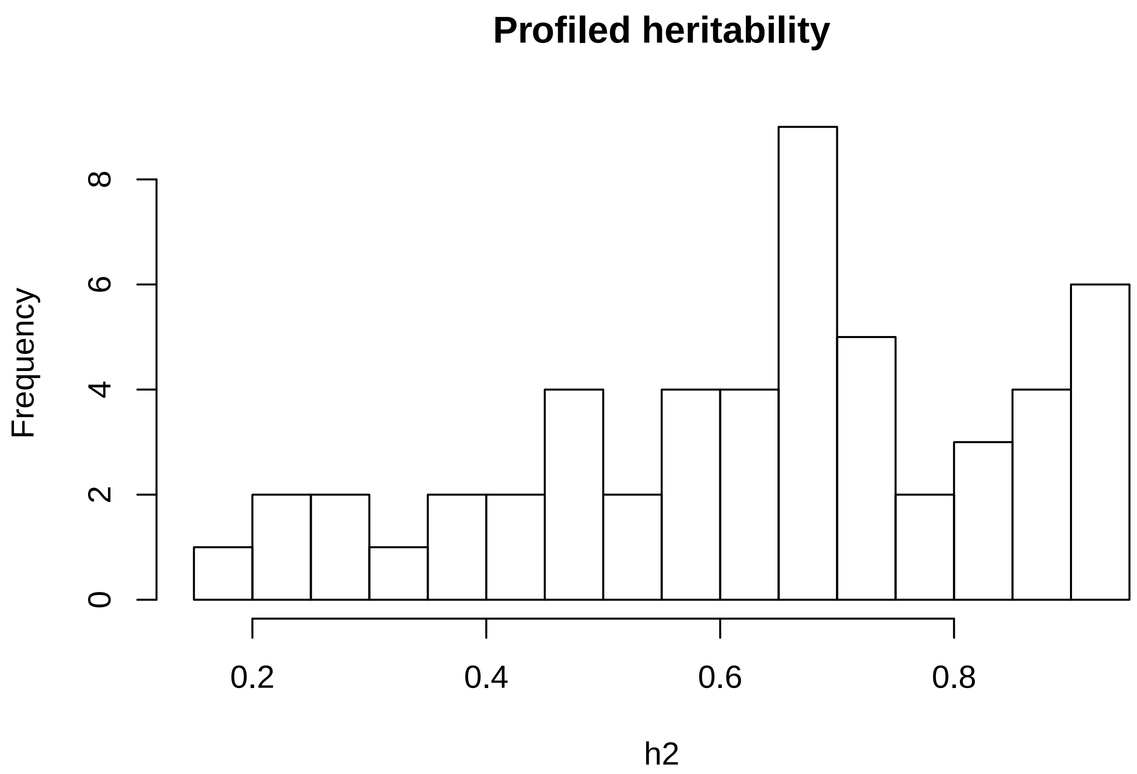

Likelihood ratio tests (LRTs)

```
# ?RLRsim::exactRLRT
m1_reduced <- update(m1, . ~ . - (1|hhid))
m1_null <- update(m1, . ~ . - (1|id))

rlrt_h2 <- exactRLRT(
  m1_reduced, # the reduced model with only the effect to be tested
  mA = m1, # the full model under the alternative
  m0 = m1_null, # the model under the null
  seed = 1
)
rlrt_h2
```

```
##
##  simulated finite sample distribution of RLRT.
##
##  (p-value based on 10000 simulated values)
##
## data:
## RLRT = 70.443, p-value < 2.2e-16
```

```
lrt_h2 <- anova(m1_null, m1)
```

```
## refitting model(s) with ML (instead of REML)
```

```
lrt_h2
```

```
## Data: phen
## Models:
## m1_null: aptt ~ age + gender + (1 | hhid)
## m1: aptt ~ age + gender + (1 | id) + (1 | hhid)
##         Df    AIC    BIC  logLik deviance  Chisq Chi Df Pr(>Chisq)
## m1_null  5 2416.2 2440.1 -1203.1   2406.2
## m1       6 2348.0 2376.7 -1168.0   2336.0 70.185      1  < 2.2e-16 ***
## ---
## Signif. codes:  0 '***' 0.001 '**' 0.01 '*' 0.05 '.' 0.1 ' ' 1
```

Summary

Heritability estimates / tests	Value
Estimate	0.56
95% CI	[0.45; 0.84]
Exact RLRT p-value	< 2.2e-16
LRT p-value	< 2.2e-16

Polygenic sex-specificity analysis

The the advanced polygenic model of sex-specificity the variance depends on gender.

```
m3 <- relmatLmer(aptt ~ age + gender + (0 + gender|id) + (0 + dummy(gender)|rid),
    phen, relmat = list(id = dkin), REML = FALSE)
VarCorr(m3)
```

```
##  Groups   Name          Std.Dev. Corr
##  id       gender1       0.82909
##           gender2       0.69727  1.000
##  rid      dummy(gender) 0.40729
##  Residual               0.52552
```

We see from the previous output of variance components that there are some sex-specific differences. To assess these difference quantitively, we will fit two null models and perform LRT:

- the genetic variances are equal;
- the genetic correlation coefficient is 1.

The later model does not make sense, as the alternative model `m3` indicates that the genetic correlation coefficient is 1.

```
m3_vareq <- relmatLmer(aptt ~ age + gender + (0 + gender|id) + (0 + dummy(gender)|rid),
    phen, relmat = list(id = dkin), vcControl = list(vareq = list(id = c(1, 2, 3))), REML = FALSE)
VarCorr(m3_vareq)
```

```
##  Groups   Name          Std.Dev. Corr
##  id       gender1       0.76577
##           gender2       0.76577  1.000
##  rid      dummy(gender) 0.17475
##  Residual               0.58803
```

The LRT suggests that we cannot conclude that there is sex-specificity.

```r
stat <- 2 * (logLikNum(m3) - logLikNum(m3_vareq))
pval <- pchisq(stat, df = 1, lower = FALSE)
pval
```

```
## [1] 0.1882005
```

The following code shows how to fit a model with a restriction that the genetic correlation coefficient is 1.

```r
m3_rho1 <- relmatLmer(aptt ~ age + gender + (0 + gender|id) + (0 + dummy(gender)|rid),
    phen, relmat = list(id = dkin), vcControl = list(rho1 = list(id = 3)), REML = FALSE)
VarCorr(m3_rho1)
```

```
##  Groups   Name          Std.Dev. Corr
##  id       gender1       0.82909
##           gender2       0.69727  1.000
##  rid      dummy(gender) 0.40729
##  Residual               0.52552
```

Summary

Null Model	LRT p-value
$\rho_g = 1$	1
$(\sigma_m)_g = (\sigma_f)_g$	0.1882005

Additional analyses

Dominance effect in addition to additive genetic and house-hold effects

A single genetic additive effect:

```r
mod1 <- relmatLmer(aptt ~ age + gender + (1|id) + (1|hhid), phen, relmat = list(id = dkin))
mod1
```

```
## Linear mixed model fit by REML ['lmerMod']
## Formula: aptt ~ age + gender + (1 | id) + (1 | hhid)
##    Data: phen
## REML criterion at convergence: 2355.299
## Random effects:
##  Groups   Name        Std.Dev.
##  id       (Intercept) 0.7270
##  hhid     (Intercept) 0.2582
##  Residual             0.5926
## Number of obs: 884, groups:  id, 884; hhid, 448
## Fixed Effects:
## (Intercept)          age      gender2
##     0.17759     -0.01687     -0.07376
```

Two genetic additive and dominance effects:

```r
mod2 <- relmatLmer(aptt ~ age + gender + (1|id) + (1|hhid) + (1|id7), phen, relmat = list(id = dkin, id
mod2
```

```
## Linear mixed model fit by REML ['lmerMod']
## Formula: aptt ~ age + gender + (1 | id) + (1 | hhid) + (1 | id7)
```

```
##    Data: phen
## REML criterion at convergence: 2353.095
## Random effects:
##  Groups   Name        Std.Dev.
##  id7      (Intercept) 0.7180
##  id       (Intercept) 0.5024
##  hhid     (Intercept) 0.2687
##  Residual             0.3375
## Number of obs: 884, groups:  id7, 884; id, 884; hhid, 448
## Fixed Effects:
## (Intercept)          age      gender2
##     0.17302     -0.01676     -0.06852
```

```
anova(mod1, mod2)
```

```
## refitting model(s) with ML (instead of REML)
```

```
## Data: phen
## Models:
## mod1: aptt ~ age + gender + (1 | id) + (1 | hhid)
## mod2: aptt ~ age + gender + (1 | id) + (1 | hhid) + (1 | id7)
##      Df    AIC    BIC  logLik deviance Chisq Chi Df Pr(>Chisq)
## mod1  6 2348.0 2376.7 -1168.0   2336.0
## mod2  7 2347.9 2381.4 -1166.9   2333.9 2.125      1     0.1449
```

Two fitting methods for gene-by-gender

```
mod3 <- relmatLmer(aptt ~ age + gender + (0 + gender|id) + (0 + dummy(gender)|rid),
    phen, relmat = list(id = dkin), REML = FALSE)
mod3
```

```
## Linear mixed model fit by maximum likelihood  ['lmerMod']
## Formula: aptt ~ age + gender + (0 + gender | id) + (0 + dummy(gender) |
##     rid)
##    Data: phen
##       AIC       BIC    logLik  deviance  df.resid
##  2353.077  2391.352 -1168.538  2337.077       876
## Random effects:
##  Groups   Name          Std.Dev. Corr
##  id       gender1       0.8291
##           gender2       0.6973   1.00
##  rid      dummy(gender) 0.4073
##  Residual               0.5255
## Number of obs: 884, groups:  id, 884; rid, 884
## Fixed Effects:
## (Intercept)          age      gender2
##     0.18150     -0.01685     -0.08525
```

```
mod4 <- relmatLmer(aptt ~ age + gender + (0 + gender|id) + (0 + gender|rid),
    phen, relmat = list(id = dkin), vcControl = list(rho0 = list(rid = 5)),
    weights = rep(1e10, nrow(phen)), REML = FALSE)
mod4
```

```
## Linear mixed model fit by maximum likelihood  ['lmerMod']
## Formula: aptt ~ age + gender + (0 + gender | id) + (0 + gender | rid)
```

```
##    Data: phen
## Weights: rep(1e+10, nrow(phen))
##      AIC      BIC   logLik deviance df.resid
##       NA       NA       NA       NA      874
## Random effects:
##  Groups   Name    Std.Dev. Corr
##  id       gender1 0.8292
##           gender2 0.6969   1.00
##  rid      gender1 0.5249
##           gender2 0.6654   0.00
##  Residual         0.8131
## Number of obs: 884, groups:  id, 884; rid, 884
## Fixed Effects:
## (Intercept)          age      gender2
##     0.18138     -0.01685     -0.08519
```

R session info

```
sessionInfo()
```

```
## R version 3.4.0 (2017-04-21)
## Platform: x86_64-apple-darwin15.6.0 (64-bit)
## Running under: OS X El Capitan 10.11.6
##
## Matrix products: default
## BLAS: /System/Library/Frameworks/Accelerate.framework/Versions/A/Frameworks/vecLib.framework/Versions
## LAPACK: /System/Library/Frameworks/Accelerate.framework/Versions/A/Frameworks/vecLib.framework/Versio
##
## locale:
## [1] en_US.UTF-8/en_US.UTF-8/en_US.UTF-8/C/en_US.UTF-8/en_US.UTF-8
##
## attached base packages:
## [1] stats     graphics  grDevices utils     datasets  methods   base
##
## other attached packages:
##  [1] solarius_0.3.0.2  gait_0.1          data.table_1.10.4
##  [4] RLRsim_3.1-3      lmerTest_2.0-33   lme4qtl_0.1.9
##  [7] boot_1.3-19       lme4_1.1-15       gridExtra_2.2.1
## [10] Matrix_1.2-9      dplyr_0.7.4       plyr_1.8.4
## [13] rmarkdown_1.5     knitr_1.15.1      devtools_1.13.1
##
## loaded via a namespace (and not attached):
##  [1] splines_3.4.0       lattice_0.20-35     colorspace_1.3-2
##  [4] htmltools_0.3.6     mgcv_1.8-17         yaml_2.1.14
##  [7] base64enc_0.1-3     survival_2.41-3     rlang_0.1.2
## [10] nloptr_1.0.4        foreign_0.8-67      glue_1.1.1
## [13] withr_1.0.2         RColorBrewer_1.1-2  bindrcpp_0.2
## [16] bindr_0.1           stringr_1.2.0       munsell_0.4.3
## [19] gtable_0.2.0        htmlwidgets_0.9     memoise_1.1.0
## [22] evaluate_0.10       latticeExtra_0.6-28 highr_0.6
## [25] htmlTable_1.9       Rcpp_0.12.13        acepack_1.4.1
## [28] scales_0.5.0        backports_1.0.5     checkmate_1.8.2
```

```
## [31] Hmisc_4.0-3         ggplot2_2.2.1       digest_0.6.12
## [34] stringi_1.1.5       grid_3.4.0          rprojroot_1.2
## [37] quadprog_1.5-5      kinship2_1.6.4      tools_3.4.0
## [40] magrittr_1.5        lazyeval_0.2.0      tibble_1.3.4
## [43] Formula_1.2-1       cluster_2.0.6       pkgconfig_2.0.1
## [46] MASS_7.3-47         assertthat_0.2.0    minqa_1.2.4
## [49] R6_2.2.1            rpart_4.1-11        nnet_7.3-12
## [52] nlme_3.1-131        compiler_3.4.0
```
